# Atmospheric, Water and Acoustic Pollution from Hydrocarbon Activities in the American Continent: A Literature Review

**DOI:** 10.3390/ijerph19159598

**Published:** 2022-08-04

**Authors:** Maylin Ordoñez-Obando, Oliver Rodas-López, Carlos Pazmiño-Uruchima, Cristopher Cañarte-Ayon, Luis Rivera-González, Kenny Escobar-Segovia

**Affiliations:** 1 Facultad de Ingenierías en Ciencias de la Tierra, Escuela Superior Politécnica del Litoral (ESPOL), Campus Gustavo Galindo Km 30.5 Vía Perimetral, Red Internacional de Investigación de Actividades Hidrocarburíferas y Energéticas (RIIAHE), Guayaquil P.O. Box 09015863, Ecuador; 2 Centro de Posgrados, Pontificia Universidad Católica del Ecuador—(PUCEM), Calle Eudoro Loor s/n y 25 de Diciembre, Portoviejo P.O. Box 130101, Ecuador; 3 Centro de Posgrados, Universidad Tecnológica Indoamérica (UTI), Quito P.O. Box 170103, Ecuador; 4 Centro de Posgrados, Universidad Técnica de Ambato (UTA), Ambato P.O. Box 180207, Ecuador

**Keywords:** petroleum contamination, atmospheric contamination, water contamination, acoustic contamination

## Abstract

Hydrocarbon activities over the years have been one of the main sources of environmental pollution, creating short and long-term impacts. This study aims to analyze the scientific production of the American continent through a bibliographic review of scientific articles published from the 1970s to the present, in order to contrast relevant scientific information about the types of pollution, water, atmospheric, and acoustic, published in the most important scientific repositories in the world, such as Scopus and Web of Science. The Prisma methodology was adopted for its development. From the plethora of articles collected, a sample of 3879 scientific articles was extracted, from which 3322 of them were excluded, leaving 557 records with remarkable information such as: country, year of publication, type of contamination, remediation if applicable, the associated oil & gas sector, and publication registration on the indexed website. It was noted that the countries with the highest scientific production are the United States, Canada, Brazil, Mexico, and Argentina. Furthermore, the Web of Science, unlike Scopus, contains more indexed publications related to the types of contamination objects relevant to this study. On the other hand, publications focused on water pollution are the only ones that come up with remediations; the rest release a smaller number of publications on these topics.

## 1. Introduction

In America, countless events related to environmental impact have been documented in different academic and scientific media, such as the case that took place in the Macondo field which developed in the Gulf of Mexico, where approximately 73 to 126 million gallons of oil were spilled into the ocean [1], the Exxon Valdez disaster in Alaska spilling 38,000 tons of oil [2], or people’s hearing loss influenced by the loud noise generated by the hydrocarbon activity [3], which produced an imbalance in the biodiversity and for humans in the ecosystems nearby. Significant advancement of knowledge will depend on possible means of access to the information and the ability to process it [4]. What was mentioned above shows how necessary it is to have access to sorted information, consolidated and validated by qualified and relevant organisms, with the purpose of acquiring a conglomerate of useful data as a robust source of information, which could be used as possible guidelines for finding solutions to the problems the reader might face.

The social development of mankind and its technologies have been increasing the pollutant loads in the environment. In other words, pollution is exponentially related to social progress over the years [5] The activities carried out in any type of industry have as a consequence an impact on the environment [6]; and taking this into consideration, pollution prevention is developed [7], the methodology for remedy depending on the scale of the damage generated in the affected area.

In the hydrocarbon industry there are operations in different stages of development in a field with the potential to extract non-renewable resources, and in three sectors: upstream (exploration, extraction, production, processing); midstream (transportation, storage), and downstream (refining, distribution to users) [8]. In addition to the aforementioned stages, there are essential activities carried out to perform the operations such as construction of access roads, well pads, treatment plants, etc. Furthermore, the extended exposure to hydrocarbon residues in areas with high maritime, air and land traffic will be reviewed [9].

The quantification of pollution problems derived from hydrocarbon activities since the 1970s in its different stages, segmentation of such a study, with relatively recent facts and action plans, are not usually developed and documented in depth. Therefore, an analysis of atmospheric, water and acoustic pollution from hydrocarbon activities in the American continent was carried out by means of a scientific bibliographic review to obtain and contrast information on the aforementioned types of pollution.

## 2. Materials and Methods

A literature review of manuscripts was carried out based on the Prisma 2020 methodology. This methodology is fundamental in the development of knowledge in a specific area through a rigorous and transparent procedure, which allows identifying, processing, and analyzing the scientific production considered relevant to the study [10]. Moreover, it allows any reader outside the field of expertise, following the research step by step by using flowcharts, to be able to achieve the same results obtained by the author of the work.

Prisma 2020 distinguishes itself from other methodologies by being a foundational piece for decision making, according to the area of application, since its information gathering abilities show great efficiency in obtaining valid answers and with a low rate of uncertainty. Additionally, the iterative nature of the method allows for some sort of self-correction throughout the process. Moreover, assuming the information is obtained only from accredited scientific sources lowers the risk of bias of interest that the author of the review could imprint on the documentation reviewed [11].

**Figure 1:**
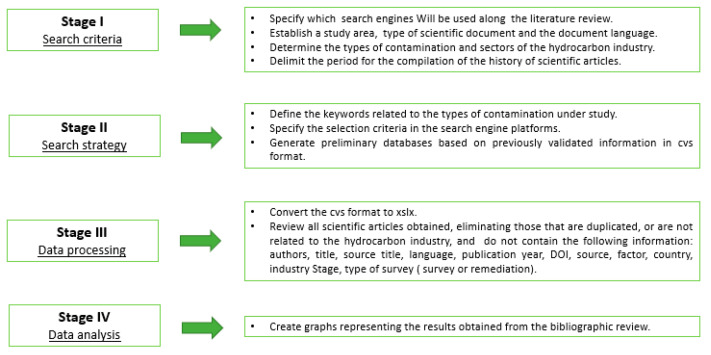
Prisma 2020 inspired flow diagram.

This present study shows a methodological process consisting of four stages (Figure 1):

### 2.1. Stage I—Selection Criteria

In this stage, the selection criteria are fundamental to ensure good research. The information with which the study will be developed is based on scientific articles obtained from reliable sources from the search engines, Scopus and Web of Science, from which the bibliographic review of the types of contamination is projected: water, atmospheric, and acoustic, in the period of time from the 1970s, due to the first world oil crisis caused by the internal struggle in the Organization of the Petroleum Exporting Countries (OPEC), to 2021 [12]. The accepted languages of the scientific articles published throughout the American continent are Spanish, English and Portuguese.

### 2.2. Stage II—Search Strategy

The selected keywords were classified into three types: water, atmospheric and acoustic, considering some stages of the hydrocarbon industry sectors as shown below (View Table 1):

The advanced bibliographic research was performed as of June 2021, due to the algorithms that are available in the virtual platforms Scopus and Web of Science through the keywords, which allow the specification of the selection criteria in order to obtain outstanding results in the csv format (comma separated values) (View Table 2).

### 2.3. Stage III—Data Processing

The data processing was established along with the selection of the scientific articles suitable for the study. It began with the conversion of the data from the csv format to xslx (file extension), which belong to Microsoft Excel for better manipulation. Then, each of the scientific articles was read and their importance was identified, matching them to the three types of contamination (water, atmospheric, acoustic), the oil & gas sector (downstream, midstream, upstream), year of publication within the study range (1970–2022), countries belonging to the American continent, sources, authors, original title, DOI, and the type of study (solution or general approach). If this information was not available, the scientific articles were excluded from the database.

### 2.4. Stage IV—Data Analysis

After being selected the articles were classified by type of contamination. Then a Microsoft Excel spreadsheet was generated with all the scientific articles; graphics were made in order to show the results in the Power BI Desktop.Ink software (Microsoft, Guayas, Guayaquil, Ecuador), due to its compatibility with the xslx format.

## 3. Results

**Figure 2:**
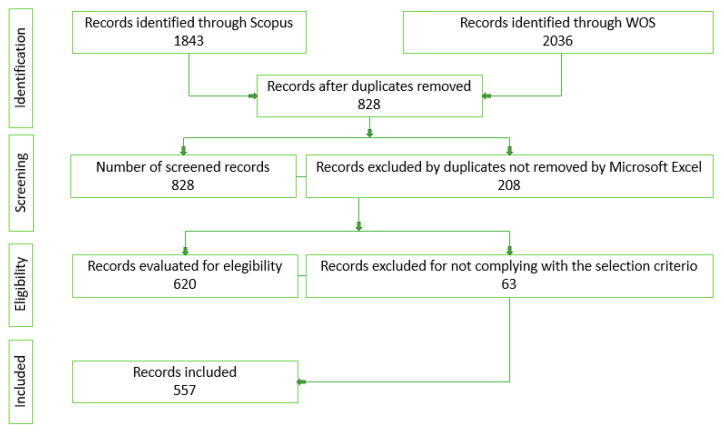
Publications of scientific articles—PRISMA 2020 inspired flow diagram.

The selection process of scientific articles relating to the hydrocarbon industry is summarized in Figure 2, which was inspired by the Prisma 2020 diagram.

### 3.1. Overview

From the searches performed, a total of 3879 scientific articles were obtained, of which 1843 were identified by Scopus and 2036 by Web of Science. Following the depuration process, 828 records were eliminated using the Microsoft Excel command to eliminate duplicates. Even though the software eliminates duplicates by taking into account the title parameter, and DOI, some repeated records were not eliminated. Therefore, it was checked once again by hand and 208 records were eliminated. This finally left 620 records of scientific articles. However, due to non-compliance with the selection criteria, 63 records were eliminated, resulting in 557 scientific articles related to the hydrocarbon industry and the different types of atmospheric, acoustic, and water pollution.

#### 3.1.1. Analysis of the Production of Scientific Articles

**Figure 3:**
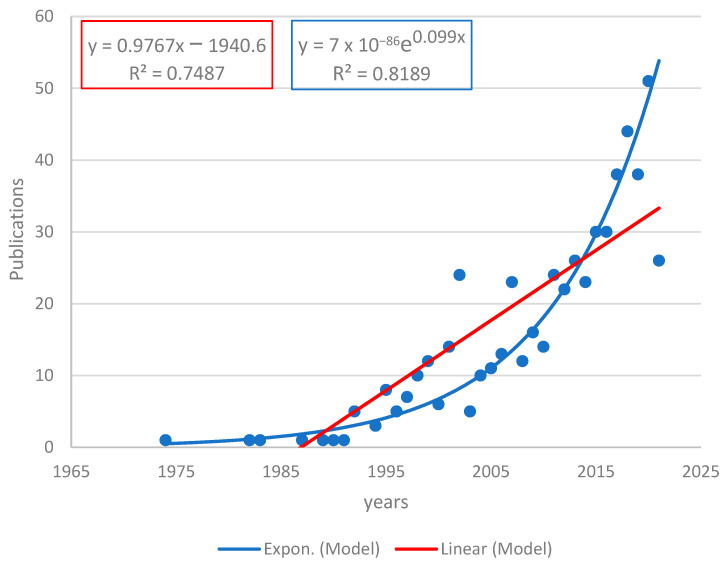
Production of scientific articles related to the hydrocarbon industry based on Scopus and WOS publications.

Figure 3 shows the production of scientific articles in the period 1970–2022, with a total of 557 articles extracted from the virtual platforms Scopus and Web of Science. An exponential growth can be noticed in the year 2005, which is based on the transience index, which allows for the evaluation of the production of authors that have only published a single work on a specific topic [13]; moreover, Price’s Law of exponential growth states that science grows at a compound interest rate, multiplied by a given amount in equal periods of time [14]. Therefore, two models were established: a linear model (y = 0.9767x − 1940.69) and an exponential model (y = 7 × 10^−86^e^0.099x^). The coefficient of determination (R^2^) shows a higher value in the exponential model (R^2^ = 0.8189) than in the linear model (R^2^ = 0.7487).

#### 3.1.2. Prevalence of Scientific Articles in the Americas Based on Separate Analysis in Scopus and Web of Science

The virtual platforms Scopus and Web of Science made it possible to review the publications of scientific articles by country, showing the prevalence of publications from different countries in the Americas, whose relationship with the hydrocarbon industry is mentioned.

**Figure 4:**
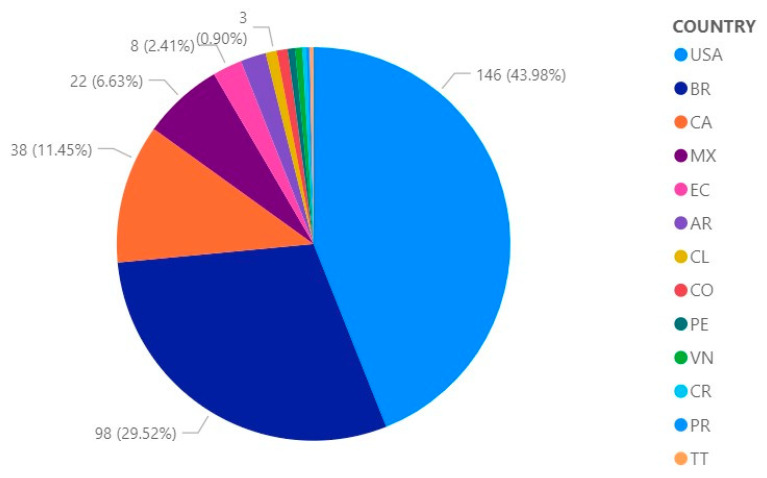
Contribution of scientific publications by country in Scopus.

Figure 4 shows the percentage contributions of the publications of scientific articles, which were obtained in Scopus, developed by the following countries: 43.98% (146) USA (United Stated of America), 29.52% (98) BR (Brazil), 11.45% (38) CA (Canada), 6. 63% (22) MX (Mexico), 2.41% (8) EC (Ecuador), 2.11% (7) AR (Argentina), 0.9% (3) CL (Chile) and CO (Colombia), 0.6% (2) PE (Peru) and VN (Venezuela), 0.3% (1) CR (Costa Rica) PR (Puerto Rico) and TT (Trinidad-Tobago).

**Figure 5:**
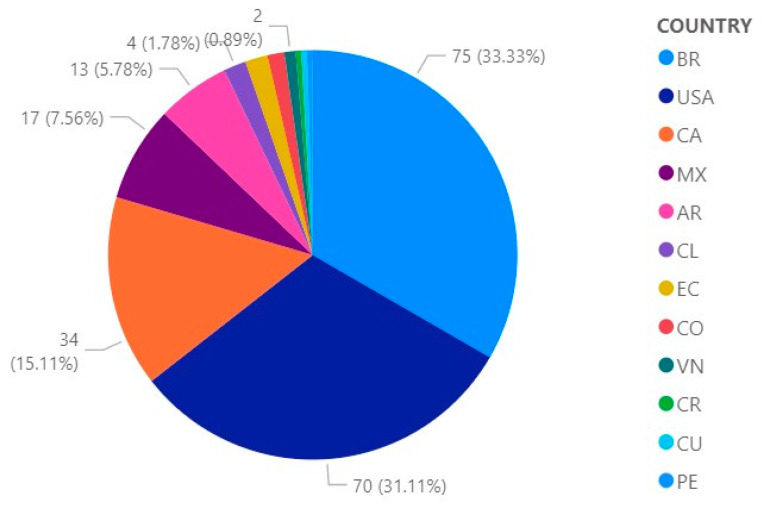
Contribution of scientific publications by country in Web of Science.

Figure 5 shows, on the other hand the percentage contributions of publications of scientific articles, as obtained from Web of Science, developed by the following countries: 33.33% (75) BR, 31.11 (70) USA, 15.11% (34) CA, 7.56% (17) MX, 5.78% (13) AR, 1.78% (4) CL and EC, 1.33% (3) CO, 0.89% (2) VN, 0.44% (1) CR, PE and CU.

#### 3.1.3. Contributions of Scientific Articles by Hydrocarbon Industry Pollution Factors Separated by Countries

**Figure 6:**
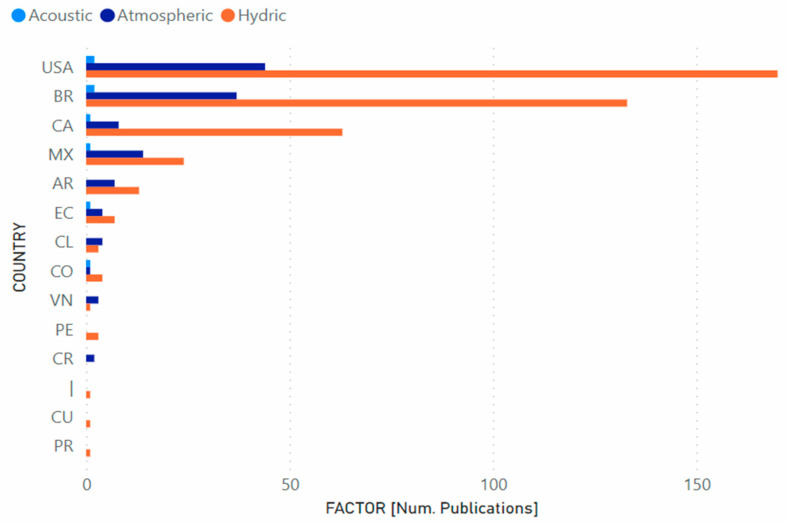
Contribution of scientific publications by country and factor.

Figure 6 shows, using bar graphs, the publication of scientific articles related to the hydrocarbon industry and the acoustic, atmospheric, and water pollution factors that have been developed, by the following countries: USA, BR, CA, MX, AR, EC, CL, CO, VN, PE, CR, CU, TT, in the virtual platforms Scopus and Web of Science.

#### 3.1.4. Number of Scientific Articles by Pollution Factors and Their Year of Publication

**Figure 7:**
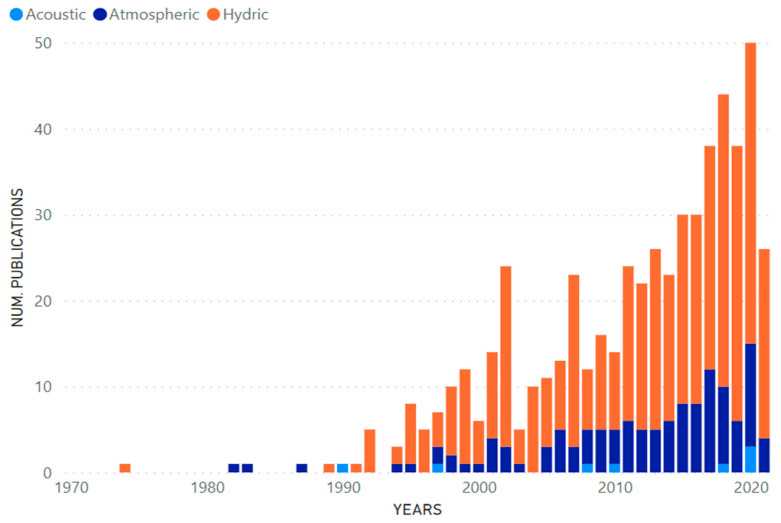
Contribution of scientific publications by years.

Figure 7 shows, using vertical bar graphs, the number of scientific articles related to the hydrocarbon industry and the acoustic, atmospheric and water pollution factors that have been developed over the years in the virtual platforms Scopus and Web of Science.

#### 3.1.5. Contributions of Scientific Articles by Pollution Factors Separated by Sector

**Figure 8:**
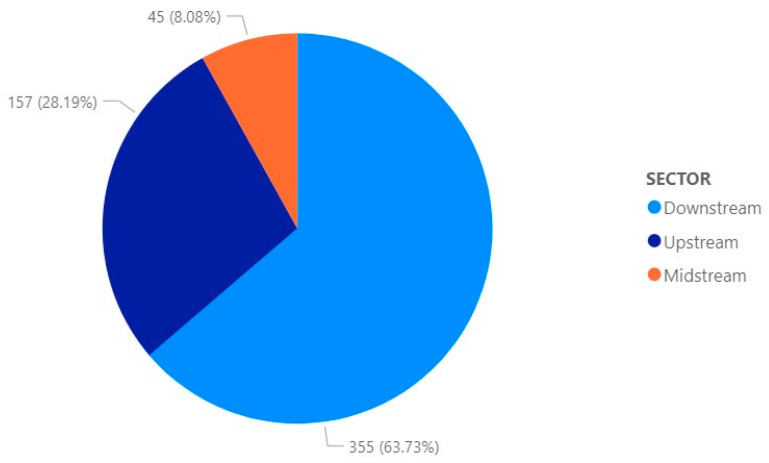
Contribution of scientific publications by sector.

Figure 8 shows the percentage contributions of the publications of scientific articles by hydrocarbon industry sector: 355 (63.73%) downstream, 157 (28.19%) upstream and 45 (8.08%) midstream.

#### 3.1.6. Number of Scientific Articles Regarding Type of Remediation to Hydrocarbon Industry Contamination by Year of Publication

**Figure 9:**
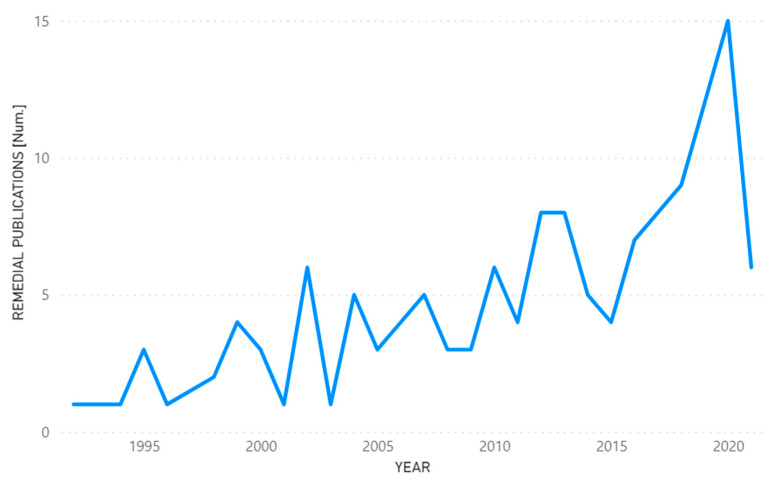
Contribution of scientific remedial publications by years.

Figure 9 shows the trend and distribution of publications of scientific articles related to environmental solutions to accidents or incidents caused by the hydrocarbon industry, and the years in which they were published.

The development of new techniques and technologies over the years in the industrial sector, especially in the hydrocarbon sector, has achieved great progress in terms of types of remediation, i.e., means to mend any damage that might be caused by the industry, directly or indirectly to the ecosystem, or even means that allow for the application of techniques to reduce the percentage of contamination to the environment, such as the problems caused by hydrocarbon bioaccumulation, having, as a baseline option, remedies that show great feasibility for fighting against large pollutants. Biosurfactants, bacteria, nanoparticles, nanofoams, willow bark, cane and mangrove residues, photo-Fenton, geotextile membranes—these are the proposals that were put forward by different authors as remediations to the different types of contamination.

**Figure 10:**
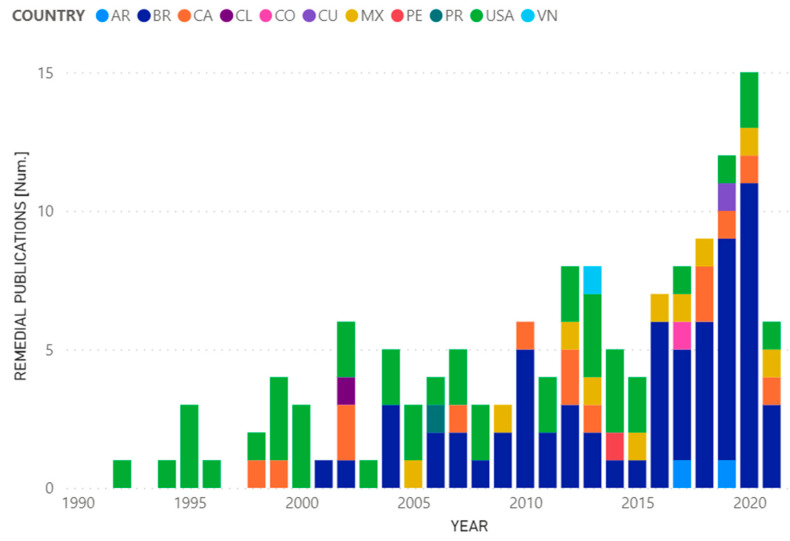
Remedial publications by years and countries.

Figure 10 shows the range of publications related to environmental remediation developed by different countries: AR, BR, CA, CL, CO, CO, CU, MX, PE, PR, USA, VN.

## 4. Discussion

The analyzed data in this study shows a bibliographic review of the relationship between the publications of scientific articles aimed at the hydrocarbon industry and the environment in the last five decades. The study focuses on the production of scientific articles from countries belonging to the American continent, where hydrocarbon activities have an impact on the ecosystem. The use of petroleum hydrocarbons in the aqueous environment often had an impact on the flora and fauna of the affected habitat, unlike soil contamination that could lead to groundwater pollution [15].

Many studies have been developed that are directly related to an environmental pollution problem due to accidents caused by hydrocarbon activities [16,17,18,19] among others, providing information from the bibliographic review and revealing that the concern for the environmental area at the American level has great importance. Due to this, the first studies were initiated in the South American region around 1994; this was related to the impact of oil activities increasing exponentially in the last 26 years [10], whilst the production of publications, as shown by the study in all America (Figure 3), with a total of 557 articles extracted from only two search engines (Scopus, Web of Science) in the period from 1970 to 2022, grew exponentially at around 9.9% in 2005.

Countries with the highest number of publications in Scopus (Figure 4) and WOS (Figure 5) are the United States, Canada, Brazil and Mexico. In a study related to the publication success of 102 nations in Scopus during the period 2005–2014, the publication success of individual countries was evaluated by tracking some scientific production indicators, their impact and also the collaboration with journals indexed to Scopus. The indexed journals show percentage growth in the period mentioned before in countries such as the United States (12.95%), Brazil (201.83%), Canada (8.40%) and Mexico (121.95%) [20]. According to an analysis of the ranking of scientific production in Latin America and the Caribbean in the period 1996–2019, the countries with the highest number of publications are Brazil, Mexico, Argentina, Chile, Colombia, Cuba and Venezuela [21]. On the other hand, the review of scientific production in the study of user experience in education in the case of Web of Science and Scopus analyzed the total publication registered in the indexing systems taken as a case study, where a sample of 194 records was obtained, resulting in the following conclusions: 80% of the records were produced in the last 5 years; 65.5% of the scientific production, articles on academic and scientific events in both Web of Science and Scopus predominated; 28.9% of the records were composed of articles published in scientific journals; 3.04% between books and chapters of scientific dissemination; and 0.50% of editorials. In addition, most of the publication belong to institutions in the United States [22].

In the last 10 years, the development of scientific articles related to water pollution resulting from oil industrial activities, has increased, such as studies that provide baselines of PAH (Polycyclic Aromatic Hydrocarbons) content that have been exposed to marine species as a result of oil spills in the ocean. An example of this is the study of fish throughout the Gulf of Mexico, where the 2010 Deepwater Horizon spill occurred; this lasted a total of seven years, in order to properly evaluate bile PAH concentrations [23].

On the other hand, but to a lesser degree the air pollution factor has omnipresent PAHs that are associated with emissions from both natural and anthropogenic sources. It was analyzed by means of a transect in the southwestern Atlantic Ocean running along the coast of Argentina from October to November 2014. The results in the gas phase levels with the highest presence found in the samples taken from the air are: phenanthrene, dibenzothiophene, fluorene and pyrene. According to the calculation of the return path of the flow of air mass and the diagnostic ratio with respect to the total concentration of PAH (gas + particles), the source is possibly influenced by something of petrogenic origin (combustion of fossil fuels) [24].

The pollution factor with the least number of publications in this study is the acoustic; however, there has been an increase in the number of publications in the last 10 years. An example of this is the study of hydrocarbon emissions from vehicles that may come from the exhaust pipe, or even from another source of gas emissions due to combustion. There is a possible correlation between vehicles being initially misidentified as having elevated exhaust pipe hydrocarbon emissions, indicating that on-road exhaust sensors could detect the presence of hydrocarbon emissions, due to evaporation, as an increase in the noise in the measurement of CO_2_ (carbon dioxide) and hydrocarbons [25].

Some studies such as “Remediation systems for the treatment of groundwater contaminated with BTEX (benzene, toluene, ethylbenzene, xylenes) and TPH (total petroleum hydrocarbons)” consist of three units. The first unit consists of the suctioning and volatilization of VOCs (volatile organic compounds); the second unit consists of an aeration tank, in order to remove volatile organic substances; and the third unit consists of a filter with half activated carbon and half rice husk ashes to remove TPH [26].

Another innovative technique is the study of the detection of water, oil and oil-in-water contamination using chirped fiber Bragg grating embedded in CYTOP (cyclic transparent optical polymer) fibers. These POF (polymer optical fiber) sensors for the detection of oil, water and oil-in-water, with their light weight, multiplexing capabilities, affinity for water and immunity to electromagnetic fields, are an ideal choice, using CFBG (chirped fiber Bragg gratings), which submerges in liquids to verify the ability to measure without taking temperature as a variable, managing to analyze the central reflected wavelength and the full width at half maximum [27].

## 5. Conclusions

After applying the different selection subject criteria, 557 scientific articles were obtained, from which the USA, with 216 publications, shows the greatest output in the development of rigorous studies of environmental incidents and accidents related to the hydrocarbon industrial pollution, closely followed by Brazil with 173 publications and, finally, followed by countries with a significant reduction in publications: Canada (72), Mexico (39), Argentina (20), Ecuador (12), Chile (7), Colombia (6), Venezuela (4), Peru (3), Costa Rica (2), Curacao (1), Puerto Rico (1), Trinidad and Tobago (1).

Furthermore, the results allowed for conclusions to be made about publications belonging to each country. For example, it was evidenced that not all the countries participating in this study have published scientific articles on the three selected types of pollutants: acoustic, atmospheric and water; and that the most recurrent publications are in the area of water pollution (425), followed by atmospheric pollution (124), and lastly acoustic pollution (8).

The remediations found in the publications of some articles present a compendium of solutions or possible solutions to problems generated by hydrocarbon activities in general. Additionally, in the last 5 years there has been an increase in the number of scientific articles published focused solely on remediating or reducing the impact on the environment.

Finally, this review and analysis can be expanded to other types of manuscripts related to pollution resulting from activities in the hydrocarbon industry, for example, effects produced on human health in nearby areas where upstream and downstream activities are carried out in the crude oil production sector. New studies can also be integrated where there is oil contamination in areas of production, storage and transportation of crude oil, among others. Therefore, this study can act as a starting point for a new article or review of articles on the broad subject of pollution caused by industrial hydrocarbon activities.

## Figures and Tables

**Table 1:** Keywords.

Keywords
atmospheric contamination AND petroleum
atmospheric pollution AND hydrocarbons
noise pollution AND hydrocarbons
noise pollution AND petroleum
water contamination AND hydrocarbons
water contamination AND petroleum
water contamination AND hydrocarbons AND drilling
water contamination AND hydrocarbons AND exploration

**Table 2:** Example of advanced search in Scopus and WOS according to selection criteria.

Examples of Advanced Search Formulation
Scopus TS = (KEY (“atmospheric contamination” AND petroleum) OR KEY (“atmospheric pollution” AND hydrocarbons) OR KEY (“noise pollution” AND hydrocarbons) OR KEY (“noise contamination” AND petroleum) OR KEY (“water contamination” AND petroleum) OR KEY (“water contamination” AND petroleum AND hydrocarbons) OR KEY (“water contamination” AND hydrocarbons AND drilling) OR KEY (“water contamination” AND hydrocarbons AND exploration) AND (LIMIT-TO (AFFILCOUNTRY, “Brazil”) OR LIMIT-TO (AFFILCOUNTRY, “Chile”) OR LIMIT-TO (AFFILCOUNTRY, “Colombia”) OR LIMIT-TO (AFFILCOUNTRY, “Venezuela”) OR LIMIT-TO (AFFILCOUNTRY, “United States”) OR LIMIT-TO (AFFILCOUNTRY, “Canada”)) AND (LIMIT-TO (LANGUAGE, “English”) OR LIMIT-TO (LANGUAGE, “Spanish”) OR LIMIT-TO (LANGUAGE, “Portuguese”))
WOS TS = ((“atmospheric contamination” AND “petroleum”) OR (“atmospheric pollution” AND “hydrocarbons”) OR (“noise pollution” AND “hydrocarbons”) OR (“noise pollution” AND “petroleum”) OR (“water contamination” AND “petroleum”) OR (“water contamination” AND “hydrocarbons” AND “drilling”) OR (“Water contamination” AND “hydrocarbons” AND (“exploration”) AND (“English*” OR Spanish* OR “Portuguese) AND (“Article”) AND (“Brazil” OR “Venezuela” OR “Chile” OR “Colombia” OR “United States” OR “Canada”))
